# Percutaneous Coronary Intervention of Native Artery Versus Bypass Graft in Patients with Prior Coronary Artery Bypass Graft Surgery

**DOI:** 10.31083/j.rcm2307232

**Published:** 2022-06-24

**Authors:** Mohamed Farag, Emmanouil S Brilakis, Gabriele L Gasparini, James C Spratt, Mohaned Egred

**Affiliations:** ^1^Cardiothoracic Department, Freeman Hospital, NE7 7DN Newcastle upon Tyne, UK; ^2^School of Life and Medical Sciences, University of Hertfordshire, AL10 9AB Hertfordshire, UK; ^3^Minneapolis Heart Institute Foundation, Minneapolis, MN 55407, USA; ^4^Department of Invasive Cardiology, Humanitas Clinical and Research Center IRCCS, 20089 Rozzano, Milan, Italy; ^5^Department of Cardiology, St George's University Hospitals NHS Foundation Trust, SW17 0QT London, UK; ^6^Newcastle University Translational and Clinical Research Institute, NE1 7RU Newcastle upon Tyne, UK

**Keywords:** PCI, CABG, vein graft, native artery, outcome

## Abstract

**Background:**

Percutaneous coronary intervention (PCI) is common in patients with prior coronary artery bypass graft surgery (CABG), however, there is limited data on the association between the PCI target-vessel and clinical outcomes. In this article, we provide a state-of-the-art overview of the contemporary management of patients with prior CABG and a clear indication for revascularization.

**Methods:**

We performed a structured literature search of PubMed and Cochrane Library databases from inception to March 2021. Relevant studies were extracted and synthesized for narrative review.

**Results:**

Twenty-six observational studies focusing on PCI of bypass graft versus native coronary artery lesions in 366,060 patients with prior CABG were included. The data from observational studies suggest that bypass graft PCI is associated with higher short- and long-term major adverse cardiac events compared to native coronary artery PCI.

**Conclusions:**

Whenever feasible, native coronary artery PCI should be the prioritized treatment for saphenous vein graft failure. Prospective randomized trials are needed to elucidate the optimal revascularization strategy for patients with prior CABG.

## 1. Introduction

Saphenous vein graft (SVG) remains the predominant conduit in patients undergoing coronary artery bypass graft surgery (CABG) despite inferior patency rates [1]. SVG failure is common with a different pathophysiology from native coronary artery disease, including compliance mismatch between artery and vein and accelerated atherosclerosis [2]. Despite better use of secondary prevention measures in patients with prior CABG, only about half of SVGs are patent at 10 years and many of those have significant atherosclerosis [3, 4]. SVG failure is associated with increased morbidity and mortality [3]. Repeat CABG poses a significant surgical challenge with increased mortality and therefore rarely performed in contemporary practice, especially with the advancements of chronic total occlusion (CTO) interventions [5]. Complex percutaneous coronary intervention (PCI) of degenerated SVGs and native coronary arteries has become a common scenario. SVG PCI accounts for approximately 6% of all PCI procedures and carries an increased risk for procedural complications, such as distal embolization and no reflow [6, 7]. This is mainly due to the fact that degenerated SVG plaques are usually soft and friable with a high content of thrombotic material and inflammatory cells. Late complications are also frequent due to in-stent restenosis and emergence of new lesions requiring multiple repeat revascularization procedures [7].

In contrast to native coronary artery lesions, drug-eluting stents (DES) do not seem to improve outcomes compared to bare metal stents in SVG lesions [6, 8, 9, 10, 11]. CABG does lead to accelerated native artery lesions progression with calcification due to changes in hydraulic factors [12], resulting in an increase in the rate as well as complexity of CTOs in this cohort [13]. Increased native artery CTO PCI complexity is associated with reduced procedural success and increased complications [14].

There is limited data to guide coronary revascularization in patients with prior CABG, as these patients are often excluded from prospective clinical trials due to multiple comorbidities and technical challenges pertaining to PCI of old grafts and complex native atherosclerotic disease. A recent meta-analysis of retrospective observational studies has shown that SVG PCI is associated with worse long-term major adverse cardiac events (MACE) compared to PCI of native coronary arteries after CABG [15]. However, this was non-randomized data and may not apply to more challenging native coronary anatomy such as CTOs [7, 16]. In this article, we review the literature comparing PCI of native coronary artery lesions with PCI of bypass graft lesions. We also review available evidence for stent choice in SVG lesions and the applicability of embolic protection device use in clinical practice. We then discuss the impact of CTO treatment on patients with prior CABG.

## 2. Methods

The study was designed according to the PRISMA (Preferred Reporting Items for Systematic Reviews and Meta-analyses) statement. We performed a structured literature search of the PubMed and the Cochrane Library databases from inception to March 2021. We used an advanced search strategy utilizing various combinations of the following MeSH terms: graft, saphenous vein, SVG, coronary artery, percutaneous coronary intervention, PCI, coronary artery bypass or CABG in the title or abstract, with no limits applied. Two reviewers independently performed the literature search and screen, with disputes resolved by consensus following discussion with other authors. Studies focused on PCI of bypass graft versus native coronary artery lesions in patients with prior CABG were selected (**Supplementary Fig. 1**). Studies were excluded if they were duplicates, single-arm studies or had indistinguishable cohorts, did not report clinical outcomes, or were conducted in the thrombolysis or balloon angioplasty era. Relevant data was extracted and synthesized for narrative review. The study outcome was short- and long-term MACE. Short-term refers to in-hospital or <30 days. Long-term refers to the longest documented follow-up.

## 3. Clinical Studies Evaluating PCI in Patients with Prior CABG

The studies evaluating PCI of bypass graft versus native coronary artery lesions in patients with prior CABG were heterogenous with conflicting results. A summary is provided in Table 1 (Ref. [16, 17, 18, 19, 20, 21, 22, 23, 24, 25, 26, 27, 28, 29, 30, 31, 32, 33, 34, 35, 36, 37, 38, 39, 40, 41]). Twenty-six studies involving 366,060 patients were included. The study-quality was assessed using the Risk Of Bias in Nonrandomized Studies of Interventions tool (ROBINS-I), as shown in **Supplementary Table 1**. All studies were observational and did not provide a clear insight into whether both treatment options were available to the operator and/or matched for the same territory of myocardial ischaemia. MACE definition and follow-up duration varied significantly between included studies (Table 1 and **Supplementary Table 2**), and therefore we felt a quantitative meta-analysis might not be reliable. Most studies reported outcomes in a small number of patients (n < 100) and have shown variable results. The revascularized bypass graft was mostly a SVG with only a few studies reporting outcomes derived also from arterial grafts [16, 17, 18, 19, 20, 21, 22], which generally represent around 2% of all bypass graft PCI [23].

**Table 1.** **Studies evaluating PCI of bypass graft versus native coronary artery lesions in patients with prior CABG**.

Study/Design	Population/Period	Follow-up	Age (year)	ACS%	DES%	Embolic protection device in BG%	CTO PCI in NA lesions%	MACE** rate%	
								Short-term†	Long-term
Meliga *et al*./2007 [24]	BG = 11	3 years	63 ± 11	29.2	100	38.4	100	BG = 0	BG = 16.1
Retrospective, single-centre registry	NA = 13							NA = 0	NA = 18.2
	Between 2002–2004							*p* = 1.00	*p* = NS
Garcia-Tejada *et al*./2009 [17]	BG = 31	1.5 years	70 ± 7	45.2	84.0	32.2	8.4	BG = 3.2	BG = 12.9
Retrospective, single-centre registry	NA = 53							NA = 3.7	NA = 15.1
	Between 2005–2006							*p* = 0.8	*p* = 0.8
Varghese *et al*./2009 [25]	BG = 63	2.5 years	66 ± 10	79.5	74.1	28.0	2	BG = 2.7	Total = 50%
Retrospective, single-centre registry	NA = 79							NA = 0	*p* = NS between groups
	Between 2003–2006							*p* = NS	
D’Ascenzo *et al*./2010 [26]	BG = 28	3 years	74 ± 8	69.8	19.0	NR	28	BG = 7.1	BG = 39.3
Retrospective, single-centre registry	NA = 25							NA = 0	NA = 28
	Between 2002–2004							*p* = NS	*p* = NS
Welsh *et al*./2010 [27]	BG = 63	3 months	68 (56–83)*	100	NR	NR	NR	NR	(Death only)
Retrospective, post-hoc analysis of RCT	NA = 55								BG = 19.0
	Between 2004–2006								NA = 5.7
									*p* = 0.050
Brilakis *et al*./2011 [23]	BG = 112,913	In-hospital	69 (60–77)*	74.0	68	NR	5.4	(Death only)	NR
Retrospective, multicentre registry	NA = 187,989							BG = 1.5	
	Between 2004–2009							NA = 0.9	
								*p *< 0.001	
Alidoosti el al./2011 [28]	BG = 63	9 months	59 ± 9	0	42.9	26.9	NR	BG = 3.2	BG = 4.8
Retrospective, single-centre registry	NA = 163							NA = 0	NA = 4.9
	Between 2003–2007							*p* = 0.077	*p* = 0.999
Gaglia *et al*./2011 [29]	BG = 191	1 year	62 ± 13	100	49.9	34.6	NR	(Death only)	BG = 36.8
Retrospective, single-centre registry	NA = 4001							BG = 14.3	NA = 24.5
	Between 2000–2010							NA = 8.4	*p* = 0.005
								*p* = 0.03	
Bundhoo *et al*./2011 [30]	BG = 60	1 year	68 ± 8	40.3	84.9	58.3	2.5	NR	BG = 21.6
Retrospective, multicentre registry	NA = 101								NA = 8.9
	Between 2005–2008								*p* = 0.048
Xanthopoulou *et al*./2011 [18]	BG = 88	2.3 years	68 ± 9	71.1	32.1	43.4	4.9	NR	BG = 43.2
Retrospective, single-centre registry	NA = 102								NA = 19.6
	Between 2004–2008								*p *< 0.001
ACROSS/2012 [31]	BG = 123	2 years	66 ± 9	36.9	83.2	NR	NR	NR	Death = 10.9
Retrospective, single-centre registry	NA = 533								MI = 10.5
	Between 2003–2008								TVF = 29.5
									*p* = NS between groups
Ho *et al*./2012 [32]	BG = 16	3 years	69 ± 14	44.0	100	6.3	0	BG = 12.5	BG = 57.9
Retrospective, single-centre registry	NA = 9							NA = 0	NA = 10
	Between 2005–2008							*p* = NS	*p* = 0.02
Nikolsky *et al*./2013 [33]	BG = 33	3 years	65 (59–74)*	100	78.0	14.0	NR	BG = 12	BG = 52
Retrospective, post-hoc analysis of RCT	NA = 50							NA = 4	NA = 30
	Between 2005–2007							*p* = 0.17	*p* = 0.04
Liu W *et al*./2013 [34]	BG = 30	2 years	62 ± 10	100	100	30.0	NR	(Death only)	BG = 26.7
Retrospective, single-centre registry	NA = 110							BG = 10	NA = 18.1
	Between 2005–2011							NA = 0	*p* = 0.21
								*p *< 0.001	
Kohl *et al*./2014 [35]	BG = 84	5 years	69 ± 12	100	NR	NR	NR	BG = 11.9	(Death only)
Retrospective, multicentre registry	NA = 104							NA = 4.8	BG = 25
	Between 2003–2012							*p* = 0.104	NA = 26
									*p* = 1.00
Liu Y *et al*./2015 [36]	BG = 75	3 years	63 ± 8	85.8	82.7	35.6	NR	NR	BG = 45.3
Retrospective, single-centre registry	NA = 190								NA = 28.4
	Between 2005–2010								*p* = 0.048
Garg *et al*./2015 [37]	BG = 25	1.7 years	65 ± 10	100	NR	NR	NR	NR	(Death only)
Retrospective, single-centre registry	NA = 22								BG = 24
	Between 2007–2012								NA = 9
									*p* = 0.253
VA-CART/2016 [38]	BG = 3616	5 years	65 (61–73)*	51.3	77.8	26.3	4.5	(Death & MI)	BG = 52.85
Retrospective, multicentre registry	NA = 7930							BG = 2.79	NA = 37.93
	Between 2005–2013							NA = 1.26	*p *< 0.001
								*p *< 0.001	
Iqbal *et al*./2016 [19]	BG = 1490	1 year	67 ± 11	100	52.1	9.4	NR	BG = 4.7	(Death only)
Retrospective, multicentre registry	NA = 1168							NA = 6.0	BG = 11.9
	Between 2007–2012							*p* = 0.18	NA = 14.5
									*p* = 0.072
Mavroudis *et al*./2017 [39]	BG = 89	3 years	73	41.4	83.0	52.8	NR	NR	(TVR only)
Retrospective, single-centre registry	NA = 103								BG = 12.5
	Between 2004–2010								NA = 3.6
									*p *< 0.001
									(Death only)
									*p* = NS between groups
ADAPT-DES/2017‡ [16]	BG = 405	2 years	69 ± 10	54.9	100	NR	5.7	BG = 2.2	BG = 18.1
Retrospective, multicentre registry	NA = 1063							NA = 1.5	NA = 8.2
	Between 2008–2010							*p* = 0.34	*p *< 0.001
Shoaib *et al*./2018 [20]	BG = 9544	1 year	71 (63–77)*	100	70.0	18.0	NR	BG = 1.52	(Death only)
Retrospective, multicentre registry	NA = 8825							NA = 2.13	BG = 7.08
	Between 2007–2014							Adjusted *p* = NS	NA = 8.29
									Adjusted *p* = NS
Liu D *et al*./2019 [21]	BG = 44	3.7 years	63 ± 8	63.0	96.8	22.7	23.0	BG = 0	BG = 25.0
Retrospective, multicentre registry	NA = 113							NA = 0	NA = 20.4
	Between 2009–2015							*p* = 1.00	*p* = 0.524
Pan-London/2020 [22]	BG = 8938	3 years	68 ± 9	46.2	87.5	15.6	28.6	BG = 3.7	(Death only)
Retrospective, multicentre registry	NA = 3703							NA = 4.3	BG = 23.8
	Between 2005–2015							*p* = NS	NA = 13.6
									*p *< 0.001
Shoaib *et al*./2020 [40]	BG = 8619	1 year	68 (61–75)*	0	67.6	NR	100	BG = 0.75	(Death only)
Retrospective, multicentre registry	NA = 2513							NA = 1.09	BG = 3.1
	Between 2007–2014							*p* = 0.1	NA = 3.5
									*p* = 0.36
Abdelrahman *et al*./2020 [41]	BG = 192	1 year	70 (63–77)*	54.6	84.5	NR	NR	NR	BG = NR
Retrospective, single-centre registry	NA = 209								NA = NR
	Between 2008–2018								*p* = 0.036 favours NA

Values are mean ± standard deviations or percentages, unless otherwise specified. * Values are median(Q1–Q3). ** The definition of major adverse cardiac events varied significantly between included studies (**Supplementary Table 2**). † Short-term refers to in-hospital or <30 days. ‡ Baseline characteristics of propensity score matching cohort in 776 patients. ACS, acute coronary syndromes; BG, bypass graft; CTO, chronic total occlusion; DES, drug-eluting stent; MACE, major adverse cardiac events; MI, myocardial infarction; NA, native artery; NR, not reported; NS, not significant; PCI, percutaneous coronary intervention; RCT, randomized controlled trial; TVF, target vessel failure; TVR, target vessel revascularization.

Brilakis and colleagues analysed a large US registry from the NCDR of approximately 300,000 prior CABG patients who underwent PCI between 2004 and 2009 and found that bypass graft PCI was performed in 37.5% of patients and was independently associated with higher in-hospital mortality compared to native coronary artery PCI [23]. Approximately, 43% of the study cohort were >10 years post CABG at the time of PCI [23]. Bypass graft PCI constituted an increasing proportion of PCI as time from CABG lengthened. In addition to increasing SVG failure post-CABG [7], progression of native coronary artery disease might have rendered native arteries less amenable to conventional PCI.

The Pan-London BCIS cohort study analysed data of 12,641 prior CABG patients who underwent PCI between 2005 and 2015 and found that bypass graft PCI was performed in 70.7% of patients and was associated with significantly higher mortality compared to native coronary artery PCI [22]. Interestingly, almost twice as many as the NCDR study had bypass graft PCI. In this analysis, unlike the NCDR study, bypass graft PCI constituted a decreasing proportion of PCI as time from CABG lengthened.

In a national cohort of US Veterans, the VA-CART study analysed data of more than 11,000 patients with prior CABG who underwent PCI between 2005 and 2013 and found that bypass graft PCI was performed in 26.6% of patients and was associated with higher incidence of short- and long-term MACE compared to culprit native coronary artery PCI, with more than double the rate of in-hospital mortality [38].

Among 2,658 patients with prior CABG who had ST-elevation myocardial infarction (STEMI) in England and Wales between 2007 and 2012, in-hospital MACE were similar between patients who underwent primary PCI to native coronary arteries compared to bypass grafts [19]. In 18,369 patients with prior CABG who had non-STEMI between 2007 and 2014, in-hospital MACE were similar between patients who underwent PCI to native coronary arteries compared to bypass grafts [20]. All-cause mortality at 30 days and 1 year was also similar between the 2 groups in both STEMI and non-STEMI cohorts.

In the case of STEMI, current guidelines recommend rapid activation of the catheterization laboratory or emergency transport to a primary PCI facility [42], which may preclude thorough review of previous ECG and operative reports in patients with prior CABG. Of note, the optimal reperfusion strategy for patients with acute SVG occlusion remains a challenge. SVGs are usually large-diameter conduits that tend to accommodate a large mass of thrombus when they are the culprit vessel [7]. Similarly, the logistic and technical challenges of dealing with severe, calcified, frequently CTO native coronary disease may not be favorable in the acute setting. Performing emergency PCI to either a SVG or a native artery in these situations is often complex and therefore physicians may choose to revascularize the easiest suspected culprit to limit the extent of infarction regardless of treatment durability.

## 4. Stent Choice in SVG Lesions

Three main mechanisms have been described for SVG failure [43]. In the early post-CABG period (first month), the main mechanism is usually acute thrombosis, which is probably due to technical and/or anatomical factors. In the late period (second-to-twelfth month), the mechanism is likely to be intimal hyperplasia, which results from the vein graft’s adaptation to higher arterial pressures. In the very late period (after 1 year), the main mechanism is often accelerated atherosclerosis, which seems to be related to the adverse characteristics of vein disease. Of note, the presence of smooth muscle and foam cells in SVG atheroma usually form unstable and fragile plaques that create a complex interaction between the endothelium and circulating platelets [44].

PCI of SVG lesions can be difficult and is frequently associated with complications [45]. Indeed, 7.7% of patients who had SVG PCI in the VA-CART study had periprocedural complications including in-hospital death and myocardial infarction [38]. Degenerated SVG lesions tend to be more lipid-rich with poorly developed or even absent fibrous cap compared to native coronary vessel lesions. Interestingly, it has been suggested that sealing even mild or moderate SVG lesions with DES does not necessarily reduce the incidence of long-term MACE compared to medical treatment alone [46, 47]. The deployment of stents in SVGs may well lead to a more enhanced inflammatory and thrombotic reaction, which may be difficult to reverse in the acute phase. Of note, adjunctive glycoprotein IIb/IIIa inhibitor administration during SVG PCI does not improve outcomes [48]. Furthermore, adjunctive intracoronary imaging tools including intravascular ultrasound and optical coherence tomography are not well studied in vein conduits. Severe SVG calcification, although uncommon, poses a high risk for stent under-expansion and therefore proper SVG lesion preparation using rotational atherectomy or lithotripsy may be required [49].

Previous studies analysed the outcomes of patients with prior CABG undergoing PCI in the era prior to stenting dominance and it was observed that the native coronary artery group had better long-term survival compared to bypass graft group [50, 51, 52]. Clinical studies in Table 1 were conducted in the stenting dominance period with more than two thirds of patients receiving DES, although likely first-generation DES. Continued technical evolution have occurred within PCI in both techniques and technology. DES implantation is proven to improve late risks when compared to bare metal stents in native coronary arteries, yet their superiority in SVG intervention is not established [6, 8, 9, 10, 11, 53, 54, 55]. Typical vein conduits diameter is frequently >4 mm, which may not particularly require DES, as the risk of restenosis is not as frequent as in relatively smaller arterial conduits. However, target vessel failure (composite of cardiac death, target vessel myocardial infarction, or target vessel revascularization) after SVG PCI occurred in approximately one in three patients during a median follow-up of 2.7 years, with no difference between bare-metal and drug-eluting stents [9]. Of note, target lesion failure (composite of cardiac death, myocardial infarction, and target-lesion revascularization) after CTO PCI was approximately 10% at 3 years follow-up [56]. This further strengthens the argument that native complex coronary artery PCI should be the prioritized treatment for SVG failure. There have been some case reports suggesting that the excess DES restenosis rate in SVGs may be related to the excessive curvature and/or mobility of DES during cardiac contractions due to the distorted anatomy leading to stent fracture and failure of local drug delivery [57, 58, 59].

## 5. Embolic Protection Devices Use in SVG Lesions

The manipulation of atherosclerotic plaque lesions with coronary wires and devices does liberate plaque contents potentially causing slow or no reflow. Clinical experience with embolic protection devices has shown that the capture and retrieval of large debris reduces periprocedural adverse events, especially in large SVGs. However, in our review, in 17 observational studies [17, 18, 19, 20, 21, 22, 24, 25, 28, 29, 30, 32, 33, 34, 36, 38, 39], and of 24,382 patients who had bypass graft PCI, only 4,487 (18.4%) had an embolic protection device used (Table 1). There are many plausible explanations for the low penetration of these devices in SVG PCI. Current devices are bulky and add complexity to procedures with an increased risk of complications. Moreover, they are not always feasible for several reasons including relatively small-calibre SVGs, inadequate distal landing zones, in-stent restenosis, and aorto-ostial lesions.

In the only available randomized study [60], embolic protection devices decreased the composite outcome of death, myocardial infarction, emergency CABG or target-lesion revascularization at 30 days (9.6% versus 16.5%). However, observational studies including data from large-scale registries are conflicting with not enough evidence to recommend the routine use of these devices [42, 61, 62]. Indeed, when embolic protection devices were used more frequently in SVG PCI, there was a higher risk of no reflow and periprocedural myocardial infarction associated with their use [38]. This however may reflect the adverse characteristics of SVG disease rather than a failure of the retrieval device. A recent meta-analysis of randomized and observational studies involving more than 50,000 patients showed no significant benefit in the routine use of these devices in contemporary real-world practice [61].

## 6. Impact of Chronic Total Occlusion (CTO) Interventions

The average overall technical success rate of recanalizing a coronary CTO is between 80 to 90% in selected series due to the advances in treatment algorithms, techniques, and equipment [63]. However, a recent meta-analysis of CTO PCI in 8131 patients showed lower technical success, higher contrast and fluoroscopy dose and higher risk of procedural complications and MACE in patients with as opposed to without prior CABG [64]. This is probably due to lesion complexity in prior CABG patients, which may restrict the success of both antegrade and retrograde approaches. As a result, in the case of multiple CTOs and as a bailout strategy, a recent consensus document suggests treating degenerated grafts instead, as multiple CTOs may limit the success of CTO PCI [65]. In 11,132 prior CABG patients with stable angina and at least 1 CTO who underwent native CTO or SVG PCI in England and Wales between 2007 and 2014, CTO PCI was performed in higher risk patients and was associated with more procedural complications (especially vessel perforation) but similar in-hospital MACE and short- and long-term mortality compared to SVG PCI [40]. The analysis demonstrates a 4-fold increase in performing CTO PCI between 2007 and 2014.

To achieve high success rate, the use of SVG as retrograde conduits seems to be safe and effective [66]. There has been recent description of acute SVG failure treated with “staged revascularization”; the culprit SVG was initially treated followed by staged revascularization of the corresponding native coronary artery CTO. Staged revascularization strategy may allow optimization of both early- and long-term outcomes [67], as CTO PCI can be challenging and often requires specialized equipment and expertise.

## 7. Discussion and Future Perspectives

The continuous refinement of PCI has contributed to a significant reduction in adverse cardiac events in recent years. In prior CABG patients, employing the percutaneous intervention strategy that provides the safest and durable revascularization with a lower risk of in-stent restenosis should be prioritized. Whenever technically feasible, treating native coronary arteries may be preferable to treating SVGs and as advocated in recent practice guidelines [68]. However, no prospective comparative data are available to support this approach and the consensus is to decide on an individual basis.

It is clear from available evidence that bypass graft PCI is associated with worse short- and long-term clinical outcomes compared to native coronary artery PCI, but there may be an equipoise between both treatments in unstable patients. To date, all studies were conducted retrospectively with all the inherent limitations of the observational design (Table 1), and therefore the results should be interpreted with great caution. These studies were subjected to bias toward patient selection, technique, and operator’s skill level. They also suffer from heterogeneity in the regime and duration of antiplatelet treatment and contemporary pharmacotherapy was not used. Moreover, PCI was undertaken in many patients using balloon angioplasty only, and hence the applicability of these studies to contemporary practice is unclear.

**Fig. 1.**
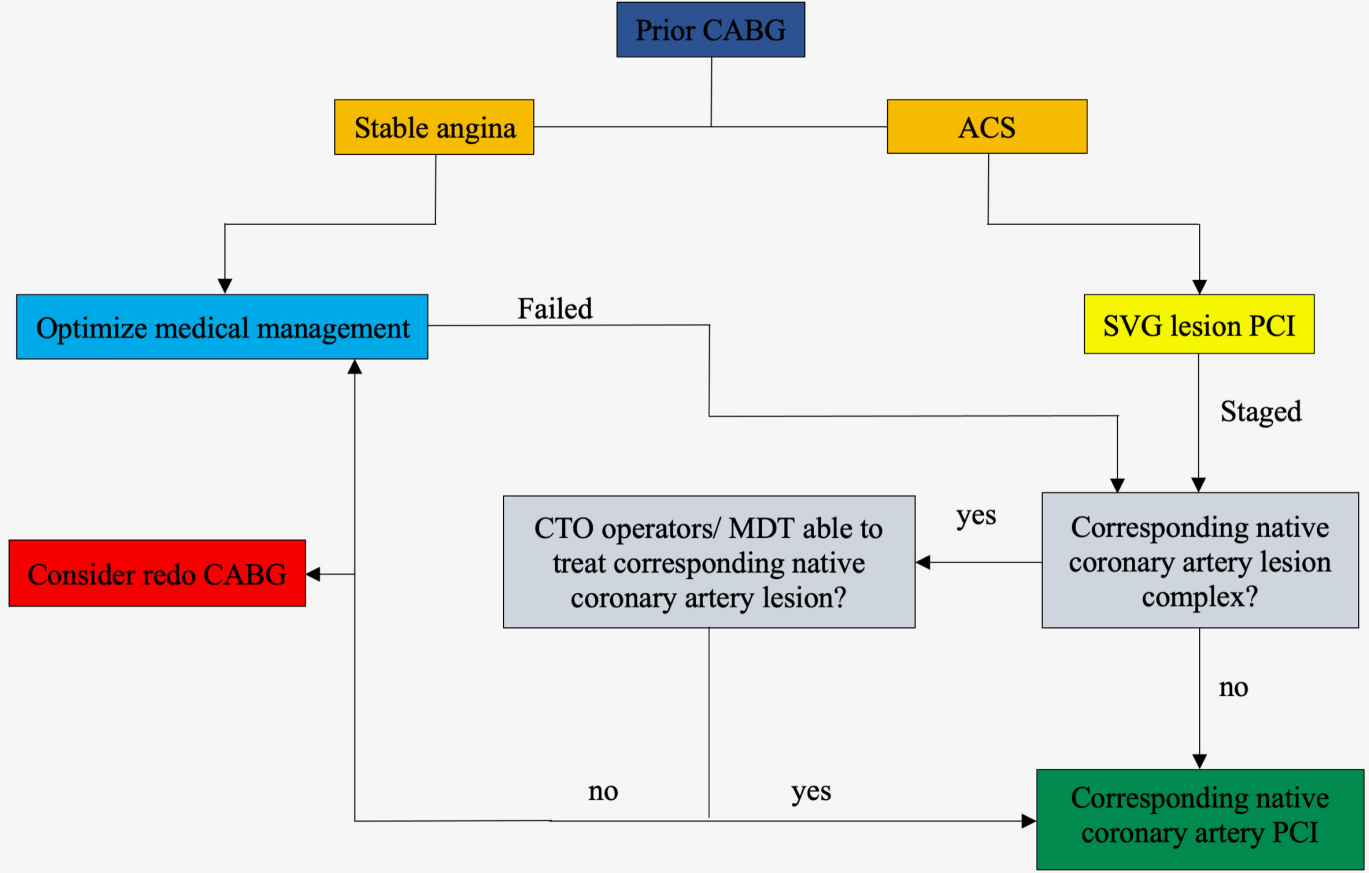
**A simplified algorithm for patients with prior CABG who have a clinical indication for PCI**. ACS, acute coronary syndromes; CABG, coronary artery bypass graft surgery; CTO, chronic total occlusion; MDT, multidisciplinary team; PCI, percutaneous coronary intervention; SVG, saphenous venous graft.

In prior CABG patients with a failed SVG and a clinical indication for revascularization, a randomized study comparing native coronary artery PCI to SVG PCI preferably with angiographic follow-up is needed. The ongoing PROCTOR (Percutaneous Coronary Intervention of Native Coronary Artery Versus Venous Bypass Graft in Patients with Prior CABG) [NCT03805048] will be the first randomized trial to investigate this complex cohort of patients. Until then, we propose a simplified algorithm for patients with prior CABG who have a clinical indication for PCI [Fig. 1].

## 8. Conclusions

In observational studies involving all-comers with prior CABG, bypass graft PCI appears to be associated with higher short- and long-term adverse cardiac events compared to native coronary artery PCI. Whenever feasible, in prior CABG patients with a clear indication for revascularization, the data from our review suggest that native coronary artery PCI should be the prioritized treatment. Prospective randomized trials are needed to elucidate the optimal revascularization strategy for such patients particularly in cases where both (SVG and native coronary artery) revascularization pathways are feasible.

## Supplementary Material

Supplementary material associated with this article can be found, in the online version, at https://doi.org/10.31083/j.rcm2307232.
