# Supplementary material for: Percutaneous Coronary Intervention of Native Artery Versus Bypass Graft in Patients with Prior Coronary Artery Bypass Graft Surgery
Source: Rev Cardiovasc Med. 2022 Jun 24;23(7):232. doi: 10.31083/j.rcm2307232 (PMC11266800; doi:10.31083/j.rcm2307232)
Supplement: Supplementary file 1 [file 2153-8174-23-7-232-s1.docx]

Records identified from:

PubMed (n = 5,863)

Cochrane Library (n = 1,073)

Records removed *before screening*:

Duplicate records removed (n = 1,036)

**Identification**

Records excluded

(n = 5,862)

Records screened

(n = 5,900)

Reports not retrieved

(n = 0)

Reports sought for retrieval

(n = 38)

**Screening**

Reports excluded:

Indistinguishable cohorts (n = 8)

Single-arm studies (n = 1)

No clinical outcomes (n = 1)

Thrombolysis (n = 1)

Balloon angioplasty only (n = 1)

Reports assessed for eligibility

(n = 38)

Studies included in review

(n = 26)

**Included**

**Supplementary Fig. 1. Study flowchart****.** *From*: Page MJ, McKenzie JE, Bossuyt PM, *et al*. The PRISMA 2020 statement: an updated guideline for reporting systematic reviews. BMJ 2021; 372: n71.

Supplementary Table 1. Risk of bias in included studies as assessed by ROBINS-I.

| Study | Confounding | Selection | Classification of intervention | Deviation from intended interventions | Missing Data | Measurement of Outcomes | Reported Result | Overall |
| --- | --- | --- | --- | --- | --- | --- | --- | --- |
| Meliga *et al.* | Moderate | Moderate | Low | Low | Low | Moderate | Low | Low |
| Garcia-Tejada *et al.* | Moderate | Low | Low | Low | Low | Low | Low | Low |
| Varghese *et al.* | Moderate | Low | Low | Low | Low | Moderate | Low | Low |
| D'Ascenzo *et al.* | Moderate | Moderate | Moderate | Low | Low | Moderate | Low | Moderate |
| Welsh *et al.* | Moderate | Low | Low | Low | Low | Low | Low | Low |
| Brilakis *et al.* | Moderate | Moderate | Low | Low | Low | Moderate | Low | Low |
| Alidoosti el al. | Moderate | Low | Low | Low | Low | Moderate | Low | Low |
| Gaglia *et al.* | Moderate | Moderate | Low | Low | Low | Low | Low | Low |
| Bundhoo *et al.* | Moderate | Moderate | Low | Low | Low | Moderate | Low | Low |
| Xanthopoulou *et al.* | Moderate | Low | Low | Low | Low | Low | Low | Low |
| ACROSS | Moderate | Low | Low | Low | Low | Moderate | Low | Low |
| Ho *et al.* | Moderate | Moderate | Low | Low | Low | Moderate | Low | Low |
| Nikolsky *et al.* | Moderate | Low | Low | Low | Low | Low | Low | Low |
| Liu W *et al.* | Moderate | Low | Low | Low | Low | Moderate | Low | Low |
| Kohl *et al.* | Moderate | Low | Low | Low | Low | Moderate | Low | Low |
| Liu Y *et al.* | Moderate | Low | Low | Low | Low | Low | Low | Low |
| Garg *et al.* | Moderate | Low | Low | Low | Low | Moderate | Low | Low |
| VA CART | Moderate | Low | Low | Low | Low | Moderate | Low | Low |
| Iqbal *et al.* | Moderate | Low | Low | Low | Low | Moderate | Low | Low |
| Mavroudis *et al.* | Moderate | Low | Low | Low | Low | Moderate | Low | Low |
| ADAPT-DES | Moderate | Low | Low | Low | Low | Moderate | Low | Low |
| Shoaib *et al.* 2018 | Moderate | Low | Low | Low | Low | Moderate | Low | Low |
| Liu D *et al.* | Moderate | Low | Low | Low | Low | Moderate | Low | Low |
| Pan-London | Moderate | Low | Low | Low | Low | Low | Low | Low |
| Shoaib *et al.* 2020 | Moderate | Low | Low | Low | Low | Low | Low | Low |
| Abdelrahman *et al.* | Moderate | Low | Low | Low | Moderate | Moderate | Low | Low |

Supplementary Table 2. Definition of major adverse cardiac events in included studies.

| Study | Major adverse cardiac events |
| --- | --- |
| Meliga *et al.* | All-cause death, nonfatal MI, or repeat revascularization |
| Garcia-Tejada *et al.* | Occurrence of death, MI, or need for a new revascularization procedure |
| Varghese *et al.* | NR |
| D'Ascenzo *et al.* | Composite of all causes of death, non-fatal MI and TVR |
| Welsh *et al.* | NR |
| Brilakis *et al.* | NR |
| Alidoosti el al. | Cardiac death, non-fatal MI (ST-elevation or non- ST elevation), and TVR or TLR |
| Gaglia *et al.* | Composite of death from all causes, Q‐wave MI, and TVR |
| Bundhoo *et al.* | Cardiac death, MI, repeat revascularization |
| Xanthopoulou *et al.* | Occurrence of cardiac death, nonfatal MI, or need for a new revascularization procedure |
| ACROSS | MI and target vessel failure, a composite of cardiac death, target vessel-related MI (ST-segment elevation or non-ST-segment elevation MI attributed to the target vessel or not attributable to another vessel) and repeated TVR, by means of CABG or PCI |
| Ho *et al.* | All causes of death, nonfatal MI, and/or repeat revascularization |
| Nikolsky *et al.* | Death, reinfarction, TVR for ischaemia, or stroke |
| Liu W *et al.* | All-cause deaths, non-fatal MI, and TVR |
| Kohl *et al.* | Death, stroke, recurrent ischaemia, or infarction |
| Liu Y *et al.* | Cardiac mortality, non-fatal MI, or TVR |
| Garg *et al.* | NR |
| VA CART | Death, or MI, or revascularization |
| Iqbal *et al.* | Composite of in-hospital mortality, in-hospital MI, and TVR |
| Mavroudis *et al.* | NR |
| ADAPT-DES | Composite of CVD, MI, or ST |
| Shoaib *et al.* | Composite of in-hospital mortality, in-hospital MI, and TVR |
| Liu D *et al.* | Combined occurrence of cardiac death, nonfatal MI, or need for a new revascularization strategy, including TLR and TVR |
| Pan-London | Composite of all‐cause mortality, PCI‐related MI (new ischemic pain with new ST elevation and elevation of enzymes whether treated with further revascularization therapy), stroke and reintervention PCI |
| Shoaib *et al.* 2020 | Composite of in-hospital mortality, in-hospital MI, and TVR, and procedural complications, including coronary perforation and major in-hospital bleeding |
| Abdelrahman *et al.* | Composite of death, MI, or TVR. |

CABG, coronary artery bypass graft surgery; CVD, cardiovascular death; MACE, major adverse cardiac events; MI, myocardial infarction; NR, not reported; PCI, percutaneous coronary intervention; ST, stent thrombosis; TLR, target lesion revascularization; TVR, target vessel revascularization.
